# Serial synchrotron and XFEL crystallography for studies of metalloprotein catalysis

**DOI:** 10.1016/j.sbi.2021.07.007

**Published:** 2021-12

**Authors:** Michael A. Hough, Robin L. Owen

**Affiliations:** 1 School of Life Sciences, University of Essex, Wivenhoe Park, Colchester, CO4 3SQ, UK; 2 Diamond Light Source, Harwell Science and Innovation Campus, Didcot OX11 0DE, UK

## Abstract

An estimated half of all proteins contain a metal, with these being essential for a tremendous variety of biological functions. X-ray crystallography is the major method for obtaining structures at high resolution of these metalloproteins, but there are considerable challenges to obtain intact structures due to the effects of radiation damage. Serial crystallography offers the prospect of determining low-dose synchrotron or effectively damage free XFEL structures at room temperature and enables time-resolved or dose-resolved approaches. Complementary spectroscopic data can validate redox and or ligand states within metalloprotein crystals. In this opinion, we discuss developments in the application of serial crystallographic approaches to metalloproteins and comment on future directions.

## Introduction

Around half of all proteins contain a metal, with approximately one quarter to a third requiring a metal to carry out their function [1,2]. The range of metals, their role and coordination geometries are extremely diverse, with chemical functionality broadened by binding of metals within organic frameworks. To understand the role of the metal, knowledge of both the three-dimensional scaffold surrounding the metal and the electronic structure of the metal is essential.

X-ray crystallography is the method of choice for atomic-level visualisation of metalloproteins [3], but suffers from the drawback that X-rays induce structural and electronic changes which often occur at the site of most interest [4,5]. Serial diffraction techniques offer a means of mitigating, or indeed eliminating, these changes providing snapshots of pristine states, for example, Refs. [6, 7, 8, 9] while also opening up the possibility of adding the fourth dimension of time. There are many challenges associated with metalloprotein serial crystallography and the electron density maps alone often cannot provide a complete picture as the electronic structure of a metal centre is an essential aspect of reactivity. In this current opinion, we focus on the role serial crystallography can play in understanding metalloprotein catalysis and the additional insight that methods complementary to the primary X-ray diffraction experiment can provide.

## Why use a serial approach to study metalloproteins?

The overwhelming majority of metalloprotein crystal structures are derived from a single crystal at 100 K and in many cases, the structures obtained are sufficient to answer the biological question in hand. Single crystals can even provide dose-resolved snapshots along redox catalytic pathways if approaches as such as multiple structures from one crystal (MSOX) are used [10,11]. The electrons that drive these reactions are symptomatic of a grand challenge in metalloprotein X-ray crystallography: radiation damage.

X-ray induced damage is most obvious in the form of a loss of diffracting power, unit cell expansion and/or increasing nonisomorphism. This global damage is often mitigated through monitoring of scaling statistics and the use of a multicrystal approach. More pressingly for metalloprotein crystallography, site-specific damage occurs at much lower doses and is more insidious. Metal centres are exquisitely prone to X-ray induced radiation damage [12] and proteins containing redox centres, such as transition metals, are highly susceptible to electronic state and structural changes as a consequence. This is particularly evident for high valent metal sites such as the Fe(IV) heme centres in peroxidases and cytochromes P450 [13], as well as in nonheme Fe(IV) centres as in isopenicillin N synthase [14]. Importantly, these site-specific changes occur at much lower doses than the loss of diffraction [∗15], providing a strong case for the incorporation of complementary methods to identify and track X-ray induced changes.

The impact of radiation damage can be minimised in two ways. At synchrotrons, the dose required for structure determination can be divided over many thousands of crystals using serial synchrotron crystallography (SSX) [∗16]. Alternatively, pulsed sources such as X-ray free-electron lasers (XFELs) can be used to collect diffraction data before damage has time to occur using serial femtosecond crystallography (SFX) [17].

Serial approaches are well suited to room temperature experiments which can allow observation of physiologically relevant conformations which may become hidden or ‘trapped out’ on cryocooling. Room temperature structures, where protein conformational dynamics may be more representative of those *in vivo*, are therefore highly desirable even if they are more challenging to obtain [18]. In particular, noncryogenic samples are important for time-resolved crystallography to allow enzyme reactions or receptor/sensor protein conformational rearrangements to occur [19].

## SSX and SFX

Serial crystallography is a relatively new technique for most of the crystallographers and techniques for sample preparation, delivery and data analysis may need to be relearnt as many thousands of crystals are typically required for an experiment. Single crystal diffraction is an important first step of a serial experiment, however, and crystallisation conditions used for obtaining single crystals can be used to determine suitable batch-like conditions for serial experiments [20,21].

Both SSX and SFX require specialist sample delivery to deliver a series of microcrystals to the beam. Widely used approaches common to both include fixed targets and high viscosity extruders, with jetting and tape-drive methods largely restricted to XFEL experiments. A comprehensive discussion of these is beyond the scope of this review but excellent recent reviews summarise this [∗16,∗22]. An alternative approach is serial femtosecond rotation crystallography (SF-ROX) where larger single crystals are cryocooled as per a conventional crystallography experiment and the crystal translated and rotated between XFEL pulses. SF-ROX has been used to obtain structures of resting-state and freeze-trapped intermediates with recent examples including peroxidase compound II [23] and copper nitrite reductase [24].

Synchrotron and XFEL serial crystallography can be seen as complementary techniques. Both offer access to room temperature data collection and both also offer access to dynamics, while serial delivery is amenable to providing novel sample conditions, for example, anaerobic environments for oxygen-sensitive metalloproteins [25]. In the context of metalloproteins, a key differentiator can be the very different radiation damage regimes in play at the different sources.

## Radiation damage in SSX and SFX

At a synchrotron, diffraction data are typically collected on timescales of milliseconds or greater and the mantra that damage is proportional to dose holds true [4]. The low doses used in serial experiments are achieved by spreading the dose required for structure determination over many crystals. It is important to note that this dose, although small, is not zero: a typical beamline can easily deposit a dose of ~30 kGy within the single, short (10 ms), exposure of each microcrystal [8]. Radiation damage within microcrystals at room temperature is only now beginning to be fully examined. Recently, De la Mora and co-workers examined site-specific X-ray dose-dependent changes including breakage of disulfide bonds and decarboxylation of acidic side chains [26].

The principle of ‘diffraction before destruction’ is an important concept that enables successful SFX data collection [27]. The femtosecond duration of pulses allows diffraction data to be collected before a crystal is destroyed and, crucially, also before any manifestation of site-specific radiation damage in the structure. Care must be taken; however, Nass et al. (2015) observed changes to the metal cluster active site of ferredoxin with high intensity 80 fs pulses [28]. An emerging consensus is that under typical experimental conditions for SFX and when using shorter pulses (≤10 fs), changes to electron density or X-ray emission spectra are generally not observed.

A fundamental difference between SFX and synchrotron approaches is the extent to which X-ray induced changes to the crystal can propagate. Very fast processes certainly occur with XFEL pulses but may not be observable in the electron density. Many of the mechanisms by which damage occurs in synchrotron experiments, for example, migration of solvated photoelectrons and other radical species formed from the ionisation of water, simply do not have time to take place during the short interaction of an XFEL pulse with a crystal.

Recent elegant work using an X-ray pump X-ray probe approach in SFX revealed time-dependent elongation of disulfide bonds with delays of as little as 35 fs between X-ray pulses [∗∗29]. It is reasonable to expect that comparable changes around metal centres would be observed, given their high ionisation cross-section and so, once again, particular care is required in the case of metalloproteins where the site of most interest is most susceptible to damage [30] even though the processes of XFEL induced damage may be very different to metal site reduction observed at synchrotron sources.

## Complementary techniques

**Figure 1:**
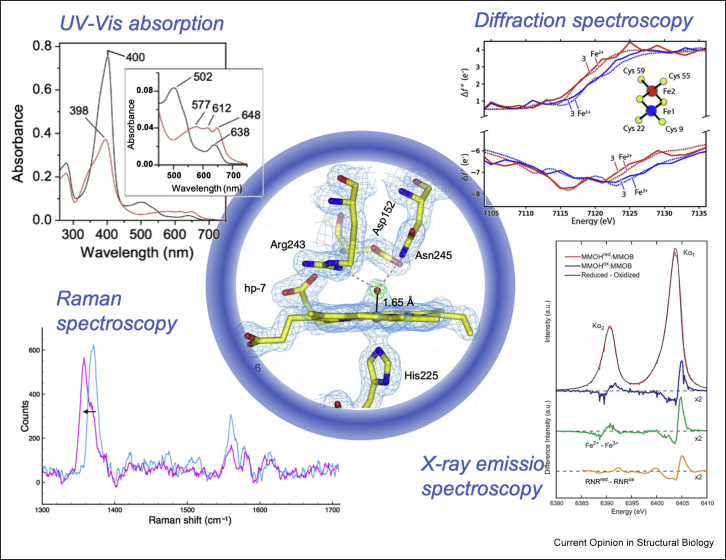
**Complementary methods for metalloprotein serial crystallography.** Clockwise from top left. UV–vis absorption spectra of a B-type dye decolorizing peroxidase (electron density shown centre) [9]. Oxidation states of iron sites in a ferredoxin were revealed using diffraction spectroscopy as shown through the use of simulated diffraction patterns [∗∗36]. Iron Kα XES of methane monooxygenase (MMOH) crystals confirming iron oxidation state [∗33]. Reprinted with permission from Ref. [33]. Copyright 2020 American Chemical Society. Single crystal resonance Raman spectra of an A-type dye-decolorizing peroxidase confirming photoreduction of the heme group during X-ray data collection [47]. Images from Refs. [9,36,47] reproduced with permission from the International Union of Crystallography.

The use of complementary spectroscopies to validate the oxidation and coordination state of metals in proteins is well established for single crystals [31], with UV–visible spectroscopy being most commonly used, alongside Raman, resonance Raman and optical fluorescence. Microcrystalline slurries may be amenable to such methods, but in particular for time-resolved experiments, spectroscopic data should ideally arise from a single microcrystal at a defined time point after reaction initiation. Some *in situ* spectroscopic methods that can help ensure that valid biological information is inferred are illustrated in Figure 1.

With the object of validating X-ray diffraction data for dynamic studies, a promising development has been simultaneous measurement of X-ray emission spectroscopy (XES) data from the individual microcrystals used for SFX [32]. Impressive recent studies have applied this approach to iron [∗33] and manganese [∗34] containing proteins. Thus far, XES has been used to identify the redox state at particular time points, but not necessarily to test in advance enzyme kinetics.

An impressive recent report describes the combination of mix-and-inject sample delivery with rapid freeze-quench electron paramagnetic resonance. Using identical sample delivery to SFX, several time points of myoglobin reacting with azide were used to establish enzyme kinetics within microcrystals [35]. This has the potential to allow time points to be precisely defined using spectroscopy before the SSX or SFX experiment.

In the near future, broad bandpass XFEL pulses also offer the promise of reconstruction of X-ray absorption edges of metal atoms within metalloproteins [∗∗36]. This will allow information on the local electronic environment of metal atoms to be derived from X-ray diffraction provided the correct X-ray energy is used, turning X-ray diffraction into a spectroscopic technique if the experimental challenges can be met.

## Dynamic metalloprotein serial crystallography

The ability to obtain time-resolved atomic resolution structures along a catalytic pathway, for both reversible and nonreversible processes, is a key aspect of serial crystallography [37]. SSX and SFX offer access to complementary timescales with, broadly speaking, SSX offering access to milliseconds and slower, and SFX to shorter time domains. Both SFX and SSX allow the study of slower processes which, depending on the triggering mechanism and system under study, may be all that are within reach.

The time resolution of a serial diffraction experiment is determined by how uniformly the reaction can be triggered throughout the crystal. MSOX, discussed briefly above, and now applied to serial experiments [38] achieved synchronous reaction initiation by using X-rays as both the pump and probe. For light-driven processes, a laser or intense LED can be used, though extreme care needs to be taken that the whole crystal volume is illuminated uniformly with as low a laser power as possible used to avoid laser-induced damage [∗39].

Although a variety of proteins are light sensitive and amenable to light-driven time-resolved studies [40], mixing crystals with substrate provides a more widely applicable means of triggering catalysis [19,∗41,^42^]. The achievable time resolution is determined by diffusion and the size of the crystals should be carefully assessed with reference to the time points being probed [43].

Photocages promise to provide the high temporal resolution of laser activation for substrate-driven reactions with biological substrate held in a photosensitive molecule soaked in ahead of the experiment [∗44].

## Some recent exemplars of metalloprotein serial crystallography

**Figure 2:**
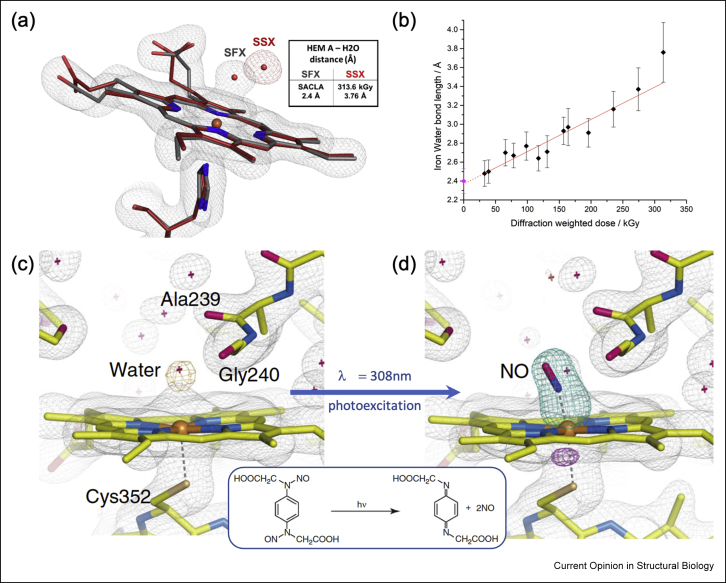
**X-ray and light-driven dynamics in metalloproteins.** Top: Following X-ray induced changes in a dye-type heme peroxidase crystal using SSX. **(a)** While differences between synchrotron and XFEL structures are well resolved for high dose SSX structures of DtpAa (a), X-ray-driven displacement of water is apparent even at low doses, which a dose-series reveal to be the first step of an X-ray-driven migration **(b)** [8]. Reproduced with permission from the International Union of Crystallography. Bottom: SFX structures of P450nor with use of a photocage to obtain intact structures of catalytic intermediates free from radiation damage. **(c)** Resting-state structure and **(d)** transient structure 20 ms after caged-NO photolysis in the absence of NADH [∗44]. Reproduced with permission from the Springer Nature.

Work on cytochrome P450NOR, a heme enzyme that reduces NO to N_2_O, used a laser-activated NO photocage [∗44]. Using a high viscosity extruder at SACLA, with careful illumination of microcrystals from two directions, a 20 ms time point was obtained, with the capture of an initial Fe–N–O intermediate (Figure 2c and d). Key to success were a microsecond release photocage, careful illumination and perhaps most important, parallel spectroscopic (visible and infrared) experiments to characterise enzyme kinetics after photocage release within microcrystals.

Fixed target SSX allows several diffraction images to be measured per crystal and this can be particularly useful for following redox phenomena. Ebrahim et al. [8] obtained 10 dose-resolved structures by measuring 10 diffraction images sequentially between each movement of the fixed target and merging images corresponding to the same dose. This revealed dose-dependent migration of a water molecule that is bound to the heme in the ferric form of a dye decolorizing peroxidase. It was possible to extrapolate the series to zero dose producing a value for the bond length identical within experimental error with the SFX structure (Figure 2a and b).

At XFELs, a key advance has been the measurement of XES spectra from microcrystals with the spectra generated from the same X-ray pulse that gives rise to the diffraction pattern. While established since 2012 [45], an increasing number of studies have used XES to track changes to oxidation states at different timepoints. In a recent example [∗∗34], manganese XES was used to follow the kinetics of the S2 to S3 transition in photosystem II at four different time points following the laser initiation. In a separate study, iron Kα XES was used to identify changes between oxidized and reduced states of soluble methane monooxygenase, demonstrating re-oxidation of the enzyme after crystals had passed through an oxygen chamber as part of sample delivery for a time-resolved experiment [∗33].

## Conclusions and summary

Serial crystallography of metalloproteins is a field of tremendous opportunity and the groundwork laid in the pioneering experiments described in this article opens the way to widespread application. The ability to obtain low-dose synchrotron, or effectively damage free XFEL, structures in combination with complementary spectroscopic probes to identify metal redox and ligation states is critical as is a range of effective means for reaction initiation for time-resolved experiments. Exciting developments applied to single crystals may become available for serial experiments in the future, for example, electrochemical control of redox state in crystals [46]. In the coming years, it is likely that time-resolved and/or dose-resolved structures, fully validated by spectroscopy will become the norm in metalloprotein structural biology. An increase in the number of serial-capable synchrotron beamlines will be a key driver for this expansion, together with the increasing availability of XFEL beamlines.

## Conflict of interest statement

Nothing declared.
